# Tongue Ultrasonography in the Screening of Severe Obstructive Sleep Apnea Syndrome—Promising Potential for Overloaded Sleep Centers

**DOI:** 10.3390/diseases12120330

**Published:** 2024-12-14

**Authors:** Milan Smiesko, Ester Jenigarova, Peter Stanko, Zsolt Kasa, Ivan Cavarga, Stefan Lassan

**Affiliations:** 1 Department of Pneumology, Phthisiology and Functional Diagnostics, Slovak Medical University and Bratislava University Hospital, 82606 Bratislava, Slovakiapete.stanko@gmail.com (P.S.); cavarga.ivan@gmail.com (I.C.);; 2 Institute of Pathophysiology, Faculty of Medicine, Comenius University, Sasinkova 4, 81108 Bratislava, Slovakia; 3 Centre of Biosciences Slovak Academy of Sciences, Institute of Animal Biochemistry and Genetics, Dúbravská cesta 9, 84005 Bratislava, Slovakia

**Keywords:** tongue ultrasonography, obstructive sleep apnea syndrome, outpatient screening, apnea–hypopnea index, polysomnography, sleep center

## Abstract

Obstructive sleep apnea syndrome (OSAS) is a frequently underdiagnosed sleep disorder marked by recurrent episodes of apnea and/or hypopnea during sleep, primarily resulting from the partial or complete collapse of the upper airway. OSAS significantly affects patients’ health and quality of life. Additionally, it is a recognized risk factor for inducing microsleep episodes during daily activities, particularly in occupations such as professional driving, where sustained attention is critical. The aim of our study was to identify an effective screening test for use in outpatient settings, capable of distinguishing patients with a severe form of OSAS. Patients who test positive with this screening tool would subsequently be prioritized for polysomnographic evaluation in a sleep laboratory. A total of 64 patients who underwent polysomnography (PSG) or polygraphy (PG) examination at our clinic were subsequently examined by USG of the tongue with measurements of tongue base thickness (TBT) and the distance between lingual arteries (DLA) during wakefulness and in a relaxed tongue position. The measurements of TBT and DLA were subsequently correlated with the apnea–hypopnea index (AHI) obtained from PSG or PG. In our cohort of patients diagnosed with severe OSAS, a TBT threshold of ≥65 mm served as an effective cutoff value. A TBT value of ≥65 mm reached an AUC value of 78.1%, sensitivity of 74.4%, specificity of 61.9%, positive predictive value of 80%, negative predictive value of 54.2% and overall accuracy of 70.3%. A DLA value of ≥30 mm in our sample of patients with severe OSAS showed an AUC of 76.5%, sensitivity of 69.8%, specificity of 71.1%, positive predictive value of 83.3%, negative predictive value of 53.6%, and overall accuracy of 70.3%. Tongue USG markers, particularly TBT and DLA measurements during wakefulness and in a relaxed tongue position, show potential as effective screening tools for identifying severe OSAS in European populations. These markers demonstrate improved accuracy over traditional screening questionnaires by reducing the likelihood of false-negative results. Patients with a positive screening should preferably be referred for polysomnography. In this way, patients with a serious illness could receive adequate therapy sooner.

## 1. Introduction

Obstructive sleep apnea syndrome (OSAS) is a frequently underdiagnosed sleep disorder marked by recurrent episodes of apnea and/or hypopnea during sleep, primarily resulting from the partial or complete collapse of the upper airway. The intermittent hypoxia associated with these repetitive apnea-arousal cycles, which restore ventilation, is a critical factor in the pathophysiological mechanisms underlying the condition [1]. OSAS has a significant impact on the health and quality of life of patients and is also a risk factor for the onset of microsleep episodes during various daytime activities. Therefore, OSAS should be promptly diagnosed, with timely implementation of appropriate therapeutic interventions [2].

The uneven distribution of sleep centers across numerous European countries has significantly contributed to prolonged wait times for polysomnography assessments, a situation further exacerbated by the COVID-19 pandemic. Available screening techniques are mostly questionnaire-based, and although they are generally sensitive enough, these techniques are not specific and have a too low NPV [2,3,4,5]. In Slovakia, the OSAS screening questionnaire (Q-OSAS) issued by the Slovak Society of Sleep Medicine (SSSM) is used [6]. Another screening tool used in pulmonology clinics is night pulse oximetry. This examination is time-consuming and requires cooperation in the home examination and subsequent evaluation by a sleep specialist [7].

The low specificity of the questionnaires may contribute to the increased demand on sleep laboratories, which are already operating at or beyond capacity. Moreover, based on our observations, relying solely on questionnaires for screening purposes often proves insufficient. This limitation arises due to the frequent occurrence of inaccurate patient self-reporting, driven by various factors. For example, professional drivers may intentionally misreport health conditions to prevent the potential loss of their driver’s license in the event of a confirmed severe illness. Another reason is the fear of continuous positive airway pressure treatment (CPAP) and its complications.

Ultrasonography (USG) is a non-invasive, available imaging examination, feasible even on an outpatient basis, relatively cheap, and without radiation exposure.

The aim of our study was to test the hypothesis that USG of the tongue could be possibly used in the screening of patients with a severe degree of OSAS. During wakefulness and in a state of tongue relaxation, tongue base thickness (TBT) and the distance between lingual arteries (DLA) emerge as ideal metrics for tongue USG screening in OSAS detection, while increased values of the TBT and DLA parameters appear to be predictors of severe OSAS [8].

## 2. Materials and Methods

### 2.1. Study Design and Participants

Our study employed a cross-sectional diagnostic study design conducted at the Department of Pneumology, Phthisiology and Functional Diagnostics, Slovak Medical University and Bratislava University Hospital, Bratislava.

A total of 64 patients with suspected sleep-related breathing disorders (SRBDs) were examined at our department, with a subsequent tongue USG examination. The tongue USG was performed before or after examination by video-polysomnography or limited cardiorespiratory polygraphy.

### 2.2. Inclusion and Exclusion Criteria

Inclusion and exclusion criteria were applied, as detailed in Table 1.

### 2.3. Data Collection

A detailed medical history was taken from the patients, focusing on symptoms and comorbidities of OSAS. In order to objectify the symptoms, patients filled out the OSAS screening questionnaire issued by the Slovak Society of Sleep Medicine (Q-OSAS) [6]. A Q-OSAS result indicated positivity with a score of 8 points or higher.

After the taking of medical history and completion of the questionnaire, the patients were objectively examined, with a body mass index (BMI) calculation. In addition to the usual status praesens generalis and localis used in internal departments, the objective examination included a specific focus on the oral cavity.

**Figure 1:**
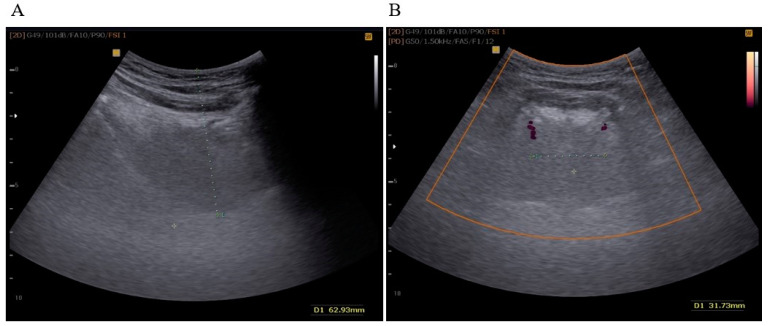
(**A**) TBT measured in the sagittal plane (green dots); (**B**) DLA measured in the coronal plane in the PD (Power Doppler) mode (green dots).

After examination of the oral cavity, the tongue USG examination was conducted with a Samsung SonoAce X8 device (convex probe 2–8 MHz). During the assessment, patients were instructed to maintain a neutral, relaxed position of the tongue. TBT was measured in the sagittal plane and DLA using the Power Doppler (PD) mode in the coronal plane (Figure 1). All examinations were performed by an experienced examiner, who is a specialist in pulmonology (M.S.), trained in performing USG of the supradiaphragmatic spaces. These procedures were carried out under the supervision of a somnologist (K.Z.). In agreement with the findings presented by Liao et al. (2016), we concur that the ultrasound methodology employed in this study is comfortable, non-invasive, and efficient for diagnosing severe OSAS [8].

TBT was expressed as the distance in millimeters between the lowest point in the convexity of the image and the highest echo point of the mucosa covering the tongue. DLA was expressed in millimeters as the distance between the Doppler image of the right and left lingual arteries at the most proximal point delineated by mandibular shadows. Initially, we set a cut-off value of TBT ≥ 60 mm and DLA ≥ 30 mm, as described in a previously published study of an Asian population by Liao et al. [8].

The results of the USG were subsequently correlated with the results of the questionnaire and the apnea–hypopnea index (AHI) value obtained through video-polysomnography (PSG) or cardiorespiratory polygraphy (PG). These assessments were performed using the in-lab PSG system SOMNOscreen^®^ plus (SOMNOmedics AG, Am Sonnenstuhl 63, D-97236 Randersacker, Germany), supplemented by home sleep testing with the Alice NightOne device (PHILIPS-Respironics, Inc., Murrysville, PA, USA). Respiratory events were scored according to the AASM criteria (American Academy of Sleep Medicine) [9]. During periods of apnea, there is a reduction in or complete cessation of respiratory amplitude exceeding 90%. In contrast, hypopnea is characterized by a partial reduction in respiratory amplitude by at least 30%. All respiratory events lasted at least 10 s and were associated with respiratory effort and a subsequent awakening response or desaturation by more than 3% of oxygen saturation. The AHI quantifies the frequency of apneic and hypopneic episodes per hour of total sleep time (TST). Patients defined as having severe OSAS had an AHI value of 30 or more per hour of TST. Patients defined as having mild OSAS had an AHI of 5 or more but less than 15 events per hour of TST. Patients classified with moderate OSAS exhibited an AHI of 15 or greater but less than 30 events per hour of TST.

### 2.4. Reproducibility

Prior to the implementation of the USG methodology in our workplace, three independent examiners (M.S., K.Z., and C.I.) evaluated this technique by applying it to the first 30 patients. Preliminary results indicated that the method consistently demonstrated high reproducibility among all investigators. All patients were examined in triplicate by an experienced ultrasonographer (M.S.), and the mean values of these measurements were used for statistical analysis. Consistent with the observations reported by Liao et al. [8], we confirm that the ultrasound methodology utilized in this study is a convenient, non-invasive, and efficient tool for diagnosing severe OSAS.

### 2.5. Statistical Analysis

GraphPad Prism version 8 software (GraphPad Software, La Jolla, CA, USA) was used for statistical analysis. For baseline data, the mean and standard deviation and standard error of the mean (SD, SEM) were used for normally distributed data and the median and range for data that were not normally distributed. Categorical variables were expressed as counts and percentages. We used a one-way ANOVA and Bonnferroni comparison test.

We employed an analysis of covariance (ANCOVA) to assess the potential influence of BMI and sex on the USG parameters of the tongue, identifying this multivariate regression model as the most suitable approach for our study design.

We conducted a receiver operating characteristic (ROC) analysis of used questionnaire-based and ultrasonographic screening tests for severe OSAS and calculated the area under the curve (AUC) to evaluate its diagnostic performance. For this purpose, XLSTAT software (version 2024.3.0, 1423, developed by Lumivero) was employed.

## 3. Results

### 3.1. OSAS Severity, Gender Distribution, BMI

**Figure 2:**
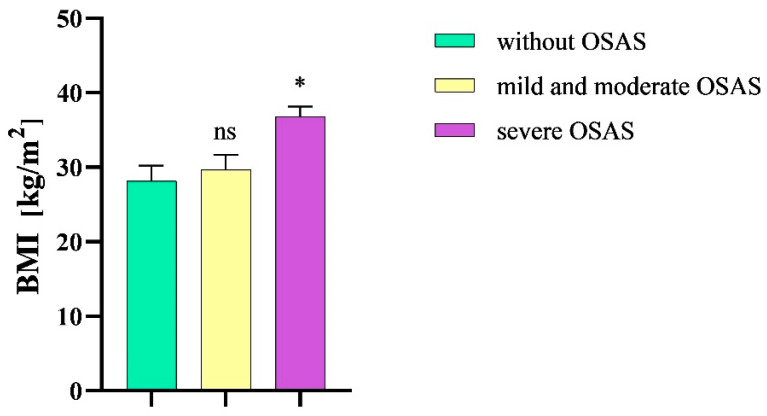
Mean body mass index in the study groups. BMI, body mass index; OSAS, obstructive sleep apnea syndrome; ns, non-significant; * *p* < 0.05.

Our analysis comprised a total of 64 participants (43 men, 21 women), where 43 individuals were diagnosed with a severe degree of OSAS, 12 participants were diagnosed with mild or moderate OSAS, and 9 participants were diagnosed with a condition other than OSAS, and they were classified as the “without OSAS” group. The age of the participants ranged from 26 to 77 years, with an average age of 53 (SD ± 13.4) years (Table 2). The mean BMI of patients without OSAS was 28.2 (SD ± 6.2) kg/m^2^. The mean BMI of patients with mild or moderate OSAS was 29.7 (SD ± 6.9) and 36.9 (SD ± 8.6) kg/m^2^ in patients with severe OSAS. We observed a statistical significance in BMI between patients with severe OSAS and patients without the given disease (*p* = 0.01) (Figure 2).

### 3.2. Questionnaire

**Figure 3:**
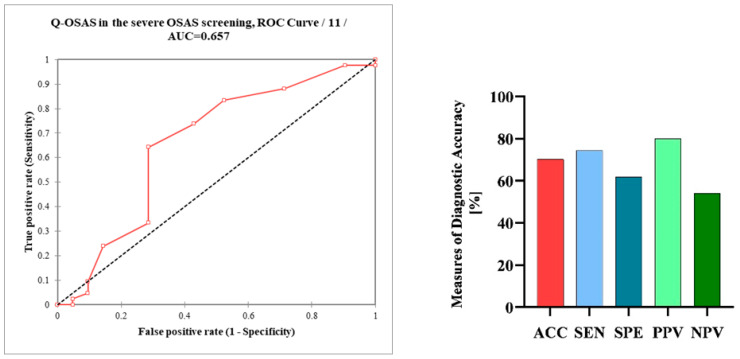
Receiver operating characteristic curve (ROC) analysis of the Q-OSAS in the screening of severe OSAS; measures of the diagnostic accuracy of the Q-OSAS in patients with severe OSAS. Q-OSAS, OSAS screening questionnaire; AUC, area under the curve; AAC, accuracy; SEN, sensitivity; SPE, specificity; PPV, positive predictive value; NPV, negative predictive value.

The Q-OSAS was positive in 74.4% of patients with severe OSAS. In patients without OSAS, the positivity of Q-OSAS significantly decreased (*p* = 0.01). Q-OSAS positivity was only numerically, not significantly, higher in patients with severe OSAS than in patients with mild or moderate OSAS (74.4% and 58.3%, respectively, *p* > 0.9). The prevalence of positive questionnaire results was not significantly different between men and women with OSAS (67.4% and 52.4%, respectively, *p* > 0.9). The Q-OSAS demonstrated an accuracy of 70.3% in patients with severe OSAS, while the sensitivity was measured at 74.4%, the specificity was determined to be 61.9%, the positive predictive value (PPV) was 80%, and the negative predictive value (NPV) was 54.2%. The calculated AUC of the Q-OSAS was 0.66 (Figure 3).

### 3.3. Ultrasonographic Parameters of the Tongue

The mean TBT value in patients with severe OSAS was 69.1 mm (SEM ± 0,9 mm), which was decreased in patients without the disease (59.2 mm, SEM ± 2.4 mm). In patients with mild or moderate OSAS, the mean TBT value was 62.2 mm (SEM ± 2.6 mm).

**Figure 4:**
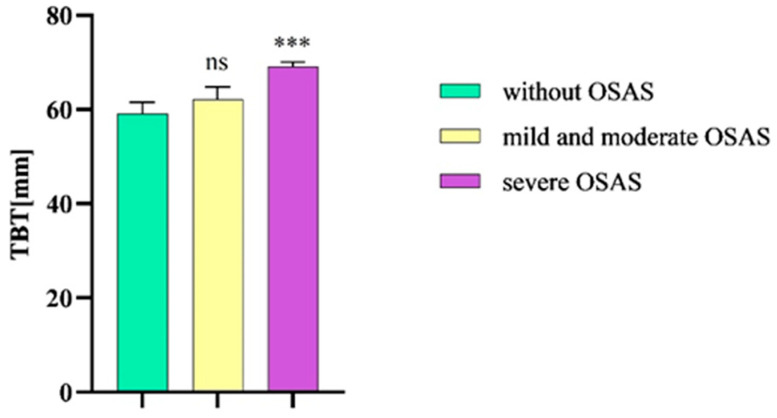
TBT in patients without OSAS compared to patients with mild and moderate OSAS and severe OSAS. TBT, tongue base thickness; OSAS, obstructive sleep apnea syndrome. Columns represent the mean of the TBT values in the individual groups of patients and their standard error of the mean (SEM); ns, non-significant; *** *p* < 0.001.

After analyzing the TBT values via an ordinary one-way ANOVA followed by a Bonferroni multiple comparison test, we observed a statistical significance between patients with severe OSAS and patients without the given disease (*p* = 0.0005). We did not detect a statistical significance of the TBT values between patients with mild and moderate OSAS and subjects without OSAS (*p* = 0.67) (Figure 4).

**Figure 5:**
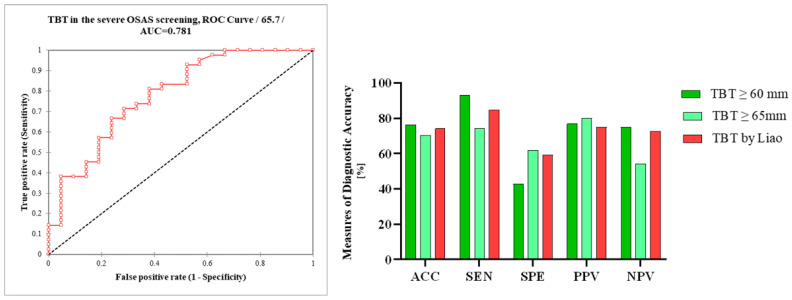
Receiver operating characteristic curve (ROC) analysis of TBT in the ultrasonographic screening of severe OSAS; differences in the measures of diagnostic accuracy between TBT ≥ 60 mm, TBT ≥ 65 mm, and TBT ≥ 60 mm using Liao et al.’s measurements. TBT, tongue base thickness; AUC, area under the curve; AAC, accuracy; SEN, sensitivity; SPE, specificity; PPV, positive predictive value; NPV, negative predictive value.

In setting the TBT cut-off value at ≥60 mm, we noted a sensitivity of 93%, specificity of 42.9%, PPV of 76.9%, and NPV of 75%, while overall accuracy was 76.6%. Due to the lower specificity, we tried to set the cut-off value of TBT at ≥65 mm for severe OSAS in our population; as a result, specificity increased to 61.9%, sensitivity dropped to 74.4%, PPV was 80%, NPV was 54.2%, and overall accuracy was 70.3%. The calculated AUC of TBT in the severe OSAS screening was 0.78 (Figure 5).

**Figure 6:**
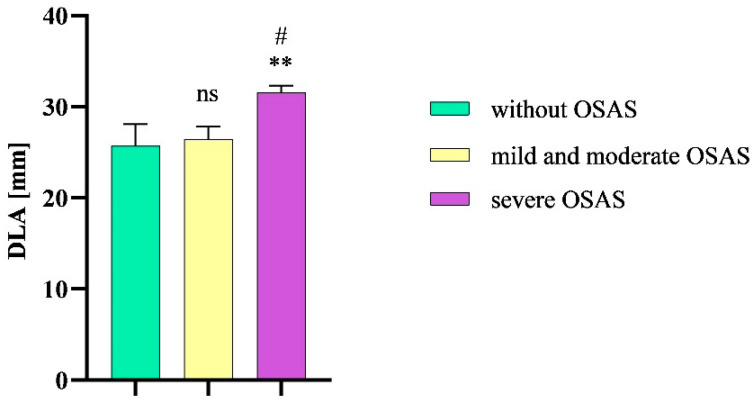
DLA in patients without OSAS compared to patients with mild and moderate OSAS and severe OSAS. Columns represent the mean of DLA values in the individual groups of patients and their standard error of the mean; DLA, distance between lingual arteries; OSAS, obstructive sleep apnea syndrome; ns, non-significant; ** *p* < 0.01 vs. without OSAS; # *p* < 0.01 vs. mild and moderate OSAS.

The mean DLA value in patients without OSAS was 25.7 mm (SEM ± 2.4 mm), while in patients with severe OSAS, the mean DLA significantly increased (31.6 mm, SEM ± 0.8 mm, *p* = 0.0148). In patients with mild or moderate OSA, the mean DLA value was 26.4 mm (SEM ± 1.4 mm). We also observed a statistical significance in DLA values between patients with severe OSAS and mild and moderate OSAS (*p* = 0.0159) (Figure 6).

**Figure 7:**
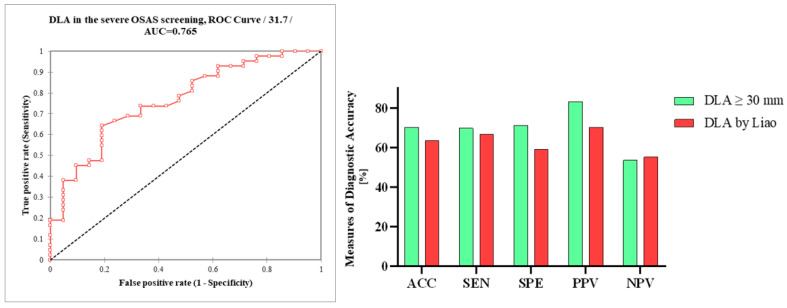
Receiver operating characteristic curve (ROC) analysis of DLA in the ultrasonographic screening of severe OSAS; differences in the measures of diagnostic accuracy between DLA ≥ 30 mm and DLA ≥ 30 mm using Liao et al.’s measurements. DLA, distance between lingual arteries; AUC, area under the curve; AAC, accuracy; SEN, sensitivity; SPE, specificity, PPV, positive predictive value, NPV, negative predictive value.

The use of a DLA threshold value of ≥30 mm resulted in a sensitivity of 69.8%, specificity of 71%, positive predictive value (PPV) of 83%, and negative predictive value (NPV) of 53.6%. The overall diagnostic accuracy was 70.3%. The calculated AUC of DLA in severe OSAS screening was 0.77 (Figure 7).

### 3.4. Multiple Regression Analysis

**Figure 8:**
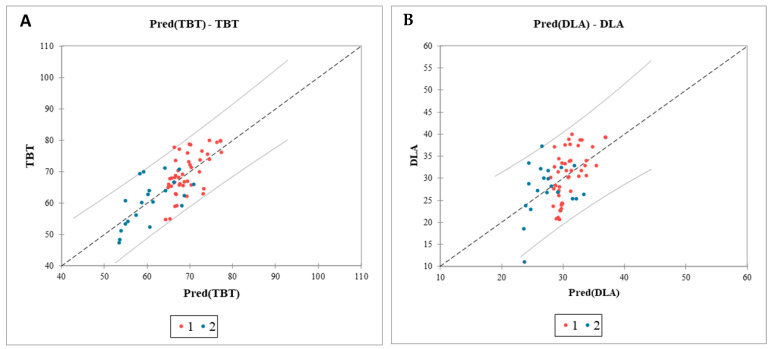
Analysis of covariance of the impact of BMI and sex on the ultrasonographic parameters of the tongue. Graph of the predicted values versus the observed values for TBT (**A**) and DLA (**B**). Confidence intervals identifying potential outliers; TBT, tongue base thickness; DLA, distance between lingual arteries; Pred, predicted values; 1 (red dots), male sex; 2 (blue dots), female sex.

We conducted an analysis of covariance (ANCOVA), selecting this multiple regression model as the most appropriate for the study design. The model’s goodness-of-fit, as indicated by the coefficient of determination (R^2^), shows that 54% of the variance in TBT and 24% in DLA, respectively, can be attributed to BMI and gender. Results from the analysis of variance and the F-test indicate a highly significant F-value (*p* < 0.0001), suggesting that both variables contribute meaningful information to the model. According to the model sum of squares (SS), each variable contributes a comparable amount of information (Figure 8).

### 3.5. Comparison of OSAS Screening Tests

**Figure 9:**
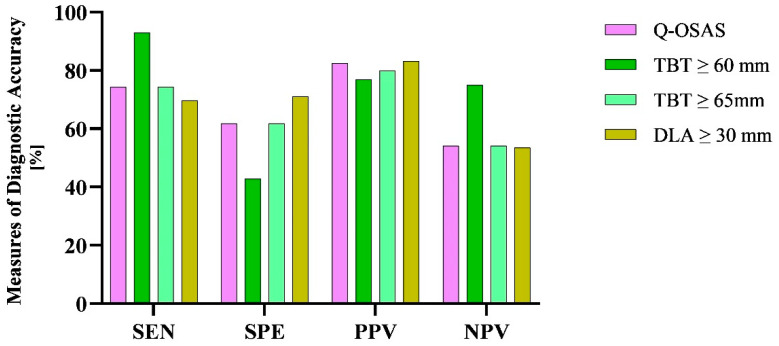
Differences in the measures of diagnostic accuracy between the USG parameters and Q-OSAS. SEN, sensitivity; SPE, specificity; PPV, positive predictive value; NPV, negative predictive value.

**Figure 10:**
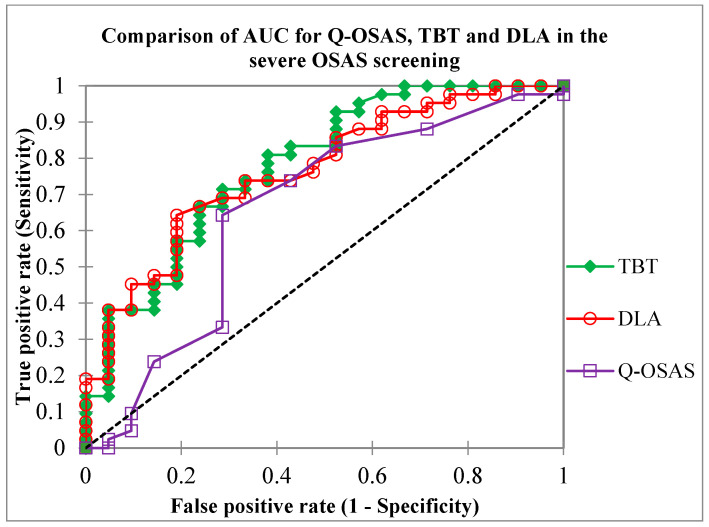
Comparison of the area under the curve (AUC) values for the TBT, DLA, and Q-OSAS metrics obtained in our study (0.78, 0.77, and 0.65, respectively). Q-OSAS, OSAS screening questionnaire; TBT, tongue base thickness; DLA, distance between lingual arteries.

Finally, we compared the results of diagnostic testing accuracy between tongue USG and a basic screening method, specifically the questionnaire. The sensitivity of both the Q-OSAS and TBT ≥ 65 mm was 74.4%. Compared to the Q-OSAS, DLA ≥ 30 mm reached a higher specificity (71% vs. 61.9%) but a slightly lower sensitivity (69.8% vs. 74.4%). TBT ≥ 60 mm gained a significantly higher NPV compared to the other conducted screening tests, while the NPV of the Q-OSAS, TBT ≥ 65 mm, and DLA ≥ 30 mm was similar (75% vs. 54.2%, 54.2%, and 53.6%, respectively). The PPV of all used screening tests was similar (82.5%, 76.9%, 80%, and 83.3%, respectively) (Figure 9). Figure 10 illustrates the comparison of the AUC for the Q-OSAS, TBT, and DLA in the screening of severe OSAS.

## 4. Discussion

OSAS stands as a nexus linking numerous prevalent health afflictions, contributing significantly to morbidity, mortality, and imposing substantial societal and economic burdens [10]. PSG, while the gold standard for OSAS diagnosing, is often inaccessible due to its high cost and limited availability [11]. Available screening techniques are mostly questionnaire-based, and although they are generally sensitive enough, these techniques are not specific and have a too low NPV [3].

Due to prolonged wait times for polysomnography examinations, attributed to a limited network of sleep centers across numerous European countries, an issue exacerbated by the COVID-19 pandemic, our objective was to identify a suitable screening tool for OSAS detection. USG is a non-invasive, available imaging examination, feasible even on an outpatient basis, relatively cheap, and without radiation exposure [12].

The tongue is one of the most common anatomical locations for an obstruction of the upper airways, blocking them through its increased volume and/or fallback position [13]. Several clinical studies demonstrated the possible use of tongue USG in the diagnosis of SRBD.

Lahav et al. observed a significant relationship between DLA and the severity of SRBD measured by PSG [14]. Shu et al. observed the transverse retropalatal diameter (RPD) and its narrowing during the Müller maneuver in patients with severe OSAS [15]. Chen et al. confirmed that the thickness of the tongue base provides information about the retroglossal airways [16].

Liao et al. conducted a study aimed at validating previously published USG parameters for the assessment of OSAS in an Asian population. They followed the DLA and the transverse RPD in the coronal plane and the TBT in the sagittal plane. Patients with severe OSAS had a significantly greater TBT and also a greater DLA in the relaxed tongue position. The cut-off value for TBT was set at 60 mm (74.2% accuracy, 84.9% sensitivity, 59.3% specificity, 75% PPV, 72.7% NPV) and 30 mm for DLA (63.6% accuracy, 66.7% sensitivity, 59.3% specificity, 70.3% PPV, 55.2% NPV). Based on their findings in the Asian population, TBT exceeding 60 mm may serve as an independent predictor of severe OSAS. Additionally, a DLA measurement greater than 30 mm may be associated with increased TBT and potentially reflects the severity of OSAS [8].

Other possibilities of using USG in OSAS diagnosis were also studied. Yu et al. utilized USG to quantify tongue fat, a factor associated with OSAS. A greater amount of adipose tissue content and its higher echo intensity was associated with a more severe AHI adjusted for age, sex, BMI, and race [17].

The study of Huang et al. showed the applicability of drug-induced sleep USG (DISU) in OSAS patients. A significantly greater mean TBT was found in drug-induced sleep than in the awake state, and the correlation between the AHI and TBT was also more significant in drug-induced sleep [18]. DLA was also significantly increased during DISU and showed a positive correlation with AHI and BMI.

Another method described by Liu et al. measured the lateral pharyngeal wall thickness (LPWT) as a distance between the medial wall of the internal carotid artery and the echogenic surface of the pharynx in an oblique coronal plane. This study revealed a positive correlation between AHI and the LPWT. LPWT showed also a positive and independent association with AHI after adjustment for age, sex, neck circumference, and BMI [19].

Along with airway parameters, some non-airway parameters, including the thickness of the carotid intimal media and mesenteric fat, diameter of the brachial artery, flow dynamics of peripheral blood, ocular blood flow, and diaphragm have also been studied for their association with OSAS through USG. Although the clinical studies evaluating these parameters have yielded inconclusive results, they highlight promising avenues for OSAS screening [12].

Imaging studies from Western countries have noted significant differences in the upper airway soft tissue structure in patients with OSAS, while the most substantial parameter found was a larger tongue volume [20,21,22]. Studies comparing the upper airway anatomy of patients with OSAS from Asia with those from Western countries have demonstrated that Asian patients have predominantly osteal rather than soft tissue restrictions [23,24].

Upon reviewing the literature, we came to the conclusion that the most suitable parameters for the USG screening of OSAS are TBT and DLA measured during wakefulness with a relaxed position of the tongue [8].

In our study on a European population, TBT and DLA significantly increased in patients with severe OSAS compared to patients without OSAS or with mild and moderate OSAS, respectively. In our study, a threshold value of TBT at 60 mm and ≥65 mm was applied. When using the cut-off value of TBT ≥ 60 mm, which was defined in a previous study conducted on an Asian population [8], we observed a better sensitivity (93% vs. 84.9%) but lower specificity (42.9% vs. 59.3%). At a cut-off of 60 mm, the test demonstrated a positive predictive value (PPV) of 76% and a negative predictive value (NPV) of 75%. When the cut-off was adjusted to ≥65 mm, the PPV increased to 80%, while the NPV decreased to 54%. These findings suggest that setting the TBT threshold value at ≥65 mm demonstrates a sufficient predictive accuracy for clinical screening purposes.

Compared to the results published by Liao et al. [8], when using the same DLA cut-off value of ≥30 mm, we observed a slightly higher sensitivity (69.8% vs. 66.7%), higher specificity (71.1% vs. 59.3%), higher PPV (83.3% vs. 70.3%), and similar NPV (53.6% vs. 55.2%). Overall accuracy was also higher (70.3% vs. 63.6%). We found significant differences in DLA between patients with severe OSAS and patients without OSAS or mild and moderate OSAS, respectively. Consequently, the DLA measurement not only proves to be an effective screening method for OSAS but also appears to be particularly well-suited for European populations, potentially even more so than for Asian populations. The use of the measurements of TBT ≥ 65 mm and DLA ≥ 30 mm effectively reduces the incidence of false-negative outcomes, which is suitable for use in the conditions of overloaded somnology centers.

The relationship between tongue morphometric characteristics and variables such as BMI, age, and gender is multifaceted [25]. This complexity arises from the potential interaction of various factors, including body composition, as well as variations in the equipment and software employed in USG and MRI assessments used in individual studies. Several studies have demonstrated a significant correlation between tongue volume and BMI [26]. Molnár et al. found that obese patients with OSAS exhibited notably larger tongue volumes, although the percentage of tongue fat did not significantly differ between groups. Despite obesity being a well-established risk factor for OSAS, no significant association was observed between tongue fat tissue and the presence of OSAS [25]. However, several studies have identified notable differences and established a correlation between AHI, tongue fat percentage, and tongue volume [17,22]. To investigate the potential relationship between BMI, gender, and measured USG parameters, a multiple regression analysis was performed. The results indicated that 54% and 24% of the TBT and DLA variability, respectively, can be explained by BMI and gender. These findings suggest an ambiguous causal relationship among the investigated factors. To elucidate this intricate network of interrelated influences, further studies are necessary, exploring various obesity phenotypes and assessing their different impacts on the tongue morphometric parameters [27].

## 5. Conclusions

USG of the tongue, especially TBT and DLA measurement during wakefulness and a relaxed position of the tongue, appears to be a suitable screening tool in patients with severe OSAS. In the European population, establishing a TBT cut-off value of ≥65 mm and a DLA cut-off value of ≥30 mm may confer an optimal clinical advantage. The use of the DLA measurement in OSAS screening seems to be even more applicable for the European population than for the Asian population.

The TBT and DLA measurements showed a similar diagnostic accuracy to the Q-OSAS questionnaire utilized in this study, while avoiding inaccurate patient self-reporting. This approach could be especially beneficial in managing patient flow in overburdened sleep centers, improving diagnostic efficiency.

### Limitations

Prior tongue surgery could potentially affect the measured USG parameters. Nevertheless, such patients were not included in our study, which constitutes a limitation of this work.

The enhanced performance of the Q-OSAS may be attributed to the setting in which it was administered; rather than being completed at a primary care facility, patients filled out the questionnaire at our specialized sleep center. This setting likely allowed for preliminary information regarding the purpose of the assessment and a basic understanding of OSAS, potentially affecting patient responses and, consequently, improving the questionnaire’s specificity.

## Figures and Tables

**Table 1:** Inclusion and exclusion criteria (PSG, polysomnography; PG, polygraphy).

Inclusion Criteria	Exclusion Criteria
Adult participants	Clinical instability or decompensation of comorbidities, as determined by a specialist
Ability to provide informed consent	History of prior neck injury
Capability to complete the questionnaire	History of prior neck surgery
Clinically stable or well-compensated physical condition	Presence of a tumor in the cervical region or tongue
The suspicion of OSAS made by a district specialist	Participant uncooperative with the investigation
Video-PSG or limited PG well-evaluable	Psychological discomfort

**Table 2:** OSAS severity and gender distribution (*n*, number of patients; BMI, body mass index).

Study Groups	Overall (*n*)	Men (*n*)	Women (*n*)	Age	BMI (kg/m^2^)
without OSAS	9	3	6	48 (SD ± 19.3)	28.2 (SD ± 6.2)
mild and moderate OSAS	12	6	6	54.6 (SD ± 11.1)	29.7 (SD ± 6.9)
severe OSAS	43	34	9	53.6 (SD ± 12.6)	36.9 (SD ± 8.6)

## Data Availability

The original contributions presented in this study are included in the article. Further inquiries can be directed to the corresponding author.
